# Neutrophil Myeloperoxidase Index in Dogs With Babesiosis Caused by *Babesia rossi*

**DOI:** 10.3389/fvets.2020.00072

**Published:** 2020-02-18

**Authors:** Anri Celliers, Yolandi Rautenbach, Emma Hooijberg, Mary Christopher, Amelia Goddard

**Affiliations:** ^1^Department of Companion Animal Clinical Studies, Faculty of Veterinary Science, University of Pretoria, Pretoria, South Africa; ^2^Department of Pathology, Microbiology and Immunology, School of Veterinary Medicine, University of California, Davis, Davis, CA, United States

**Keywords:** ADVIA 2120, *Babesia*, cytokines, dog, interleukin, MPO, MPXI, neutrophil

## Abstract

Babesiosis caused by the virulent tick-borne hemoprotozoan, *Babesia rossi*, results in a marked systemic inflammatory host response in dogs. Neutrophils form part of the innate immune response and contains myeloperoxidase (MPO) as the predominant component of the neutrophil lysosomal protein in azurophilic granules. The neutrophil myeloperoxidase index (MPXI), determined on the ADVIA hematology analyzer, is a quantitative estimate of intracellular MPO content. Objectives of this study were to: (a) compare MPXI in dogs with babesiosis with healthy control dogs; (b) compare MPXI in dogs that died from babesiosis with dogs that survived and controls; and (c) correlate the MPXI with the previously determined segmented and band neutrophil count and cytokine concentrations in dogs with babesiosis. Data for 140 dogs naturally infected with *B. rossi* and 20 healthy control dogs were retrospectively evaluated. Neutrophil counts and MPXI were determined on an ADVIA 2120 analyzer. Cytokine concentrations [interleukin (IL)-2, IL-6, IL-8, IL-10, IL-18, granulocyte-macrophage colony stimulating factor (GM-CSF), and monocyte chemo-attractant protein-1 (MCP-1)] were determined using a canine-specific multiplex immunoassay. The mortality rate of the *Babesia-infected* dogs was 11% (15/140). MPXI was significantly higher in *Babesia-infected* dogs (*P* = 0.033), and in *Babesia-infected* non-survivors (*P* = 0.011), compared with healthy control dogs. In *Babesia-infected* dogs a significant positive correlation was found between MPXI and IL-10 (*r* = 0.211, *P* = 0.039) and a significant negative correlation was found between MPXI and IL-8 (*r* = −0.350, *P* < 0.001). In *Babesia-infected* non-survivors, significant positive correlations were found between MPXI and IL-2 (*r* = 0.616*, P* = 0.033), IL-6 (*r* = 0.615, *P* = 0.033), IL-18 (*r* = 0.613, *P* = 0.034), GM-CSF (*r* = 0.630, *P* = 0.028), and MCP-1 (*r* = 0.713, *P* = 0.009). In *Babesia-infected* survivors, a significant negative correlation was found between MPXI and IL-8 (*r* = −0.363, *P* = 0.001). MPXI was correlated with pro-inflammatory cytokines in *Babesia-infected* dogs that died. The potential of MPXI as a novel marker of inflammation and prognosis in dogs infected with *B. rossi*, thus warrants further investigation.

## Introduction

*Babesia rossi* is a hemoprotozoan parasite, transmitted by *Haemophysalis elliptica* ticks, and is considered the most pathogenic of the large canine babesias (1, 2). The severity of the disease has been reported to be due to an exuberant and ineffective immune response that sometimes results in lethal collateral organ damage (3–6). Recent studies have confirmed the presence of a marked pro-inflammatory response in dogs infected with *B. rossi*, of which the severity was correlated with patient outcome (7, 8). The study by Goddard et al. (7), of which some of the published data will be used in our study, reported that concentrations of interleukin (IL)-2, IL-6, IL-8, IL-10, IL-18, monocyte chemotactic protein (MCP)-1, and granulocyte-macrophage colony stimulating factor (GM-CSF), were correlated with the severity of the pro-inflammatory response and outcome, in *B. rossi*-infected dogs. The study also showed that both C-reactive protein (CRP) and serum amyloid A were significantly increased in the *Babesia*-infected group (7). Similarly, the study by Leisewitz et al. (8) showed that IL-6, MCP-1, as well as tumor necrosis factor (TNF)-α were significantly higher in *B. rossi*-infected non-survivors compared to those that survived (8). Interestingly, IL-8 was found to be negatively correlated with disease severity for both studies, with the control group having higher concentrations than either survivors or non-survivors (8).

The ADVIA 2120, an automated hematology analyzer, uses intracellular myeloperoxidase (MPO) content and cell volume to determine the automated differential leukocyte count (9, 10). Quantitative changes for intracellular neutrophil MPO staining can be determined through calculation of the myeloperoxidase index (MPXI), as well as by observing the staining intensity (peroxidase activity, *x*-axis) and light scatter (cell size, *y*-axis), that determine the location of cells on the generated peroxidase scattergram (9, 10). Cluster analysis software gates these cell populations based on the above mentioned variables (11). In humans, MPO content is influenced by the age, toxicity, and degranulation of the neutrophil (12). MPO is predominantly produced in promyelocytes and mature neutrophils function solely to store MPO (13). The MPXI thus seems to be regulated by a balance between MPO production in promyelocytes in the bone marrow and the use of peripheral blood neutrophils during inflammation and the respiratory burst reaction (13, 14). In humans, MPXI has been investigated as a marker of inflammation (13, 15). One study reported that MPXI was higher in people with sepsis compared to those with systemic inflammatory response syndrome (SIRS) due to non-infectious causes, but in most other studies it was found to be decreased in people with sepsis and severe inflammatory reactions (13, 16, 17). Lower MPXI values are reported to indicate neutrophil activation and thus systemic inflammation (18).

There is a dearth of research on MPXI in domestic animals. A study in horses with systemic inflammation correlated low MPXI values with an increased risk of mortality, especially when it remains unchanged 24 h after therapeutic intervention (19). Neutropenic septicemic foals were found to have an increased MPXI (20). In experimental canine monocytic ehrlichiosis, the MPXI was decreased 14 and 21 days post-infection and was attributed to the continual neutropenia seen with ehrlichiosis, or defects in the maturation process of neutrophils, resulting in a lower MPO content (21). Diseases causing severe leukocyte consumption can lead to acquired MPO deficiency (MPOD) in dogs (10). To the author's knowledge, MPXI has not yet been investigated in dogs with babesiosis. Since both the innate and acquired immune systems are involved in babesiosis (22), investigating the changes in MPXI in neutrophils of dogs suffering from this disease, may broaden our understanding of the host response and might aid in the prognostication of these cases.

The objectives of this study were to retrospectively compare MPXI in dogs infected with *B. rossi* to that in healthy control dogs and to establish the correlation of MPXI with the severity of the host inflammatory response, using cytokine concentrations and neutrophil counts. We hypothesize that MPXI will be decreased in *Babesia*-infected dogs compared to healthy controls and that MPXI will be correlated with pro-inflammatory and immune-modulating cytokines.

## Materials and Methods

### Study Design

This was a retrospective observational study that evaluated the admissions records of MPXI in dogs with babesiosis caused by *B. rossi* and healthy control dogs, generated on an automated hematology analyzer (ADVIA 2120, Siemens, Munich, Germany), using multispecies software and the canine species setting at the Clinical Pathology laboratory, Onderstepoort Veterinary Academic Hospital. The study was approved by the Faculty Research Ethics committee (REC041-18).

### Study Population

Data generated by the ADVIA 2120 were retrospectively evaluated on two study population cohorts: 96 *Babesia*-infected dogs and 15 healthy control dogs, collected between October 2011 to April 2013 as part of the first study cohort (7), as well as 44 *Babesia*-infected dogs and five healthy control dogs, collected during January to December 2014 as part of the second study cohort (6). Both studies were approved by the University of Pretoria's Animal Ethics committee (V055-11 and V091-13, respectively). Owner consent was acquired for enrolment of all cases in both studies. The objective of the first study was to investigate cytokine concentrations in *B. rossi*-infected dogs and its association with disease outcome (7). The objectives of the second study were to investigate changes in selected peripheral blood lymphocytes in dogs infected with *B. rossi* at various time points and to determine correlation with the disease severity (6). The second study cohort did not include cytokine analysis; however, it was included to increase the number of control cases and dogs that had died.

For both studies, inclusion criteria included: dogs of either breed or gender; older than 12 weeks of age; weighing more than 5 kg (first cohort) or more than 3 kg (second cohort); and with a demonstrable parasitemia on blood smear, that was confirmed as *B. rossi* by polymerase chain reaction (PCR) and reverse line blot (RLB). Criteria for exclusion for the first study cohort were RLB-PCR confirmation of co-infection with *B. vogeli*, the only other large *Babesia* spp. found in the area, or *Ehrlichia canis*; euthanasia for reasons other than a poor prognosis; evident comorbidities such as existing inflammatory or infectious, cardiac, neoplastic or traumatic conditions; or treatment with anti-inflammatory therapy at, or within 4 weeks prior to presentation. Criteria for exclusion for the second study cohort were RLB-PCR confirmation of co-infection with *B. vogeli* or *E. canis;* concurrent infections, wounds or indications of trauma; vaccination, glucocorticoid therapy or any unrelated metabolic disease within 4 weeks prior to presentation.

Patients in either study cohort received the standard therapy for canine babesiosis, which involved treatment with diminazene aceturate (Berenil RTU 0.07 g/mL, Intervet, Kempton Park, South Africa) at 3.5 mg/kg and transfusion with packed red blood cells and/or intravenous fluids therapy as indicated. Any complications were treated as deemed appropriate by the attending clinician. Outcome was noted as short-term survival (i.e., until discharge from hospital), or death/euthanasia due to poor prognosis during hospitalization.

The controls included 20 healthy, client-owned dogs admitted for blood donation or routine ovariohysterectomy or castration. The control dogs were regarded to be healthy based on history, a complete physical examination, peripheral blood smear evaluation, complete blood count (CBC), complete serum biochemistry profile as well as RLB-PCR assay to exclude infection with any hemoparasites. The *Babesia*-infected study population was further divided based on outcome (survivors and non-survivors).

### Sample Collection and Laboratory Methods

Blood was collected from the jugular vein in EDTA and serum Vacutainer (BD Biosciences, New Jersey, United States) tubes at presentation, prior to any treatment, for a CBC on the ADVIA 2120 and a complete serum biochemical profile (total serum proteins, albumin, globulin, alanine aminotransferase, alkaline phosphatase, basal bile acids, total bilirubin, urea, creatinine, sodium, potassium, chloride, and ionized calcium). Blood smear evaluation was performed on all dogs by experienced hematology technologists and a 100-cell manual differential leukocyte count was performed. The generated peroxidase scattergrams were evaluated for each sample by an experienced clinical pathologist (AG), to confirm correct separation of the neutrophil population. In the first study cohort, blood was also collected in serum Vacutainer tubes for determination of cytokine concentrations (IL-2, IL-6, IL-8, IL-10, IL-18, GM-CSF, and MCP-1) (7). The cytokine concentrations were determined using a validated commercially available canine-specific multiplex immunoassay (MILLIPLEX MAP Canine Cytokine/Chemokine Magnetic Bead Panel CCYTO-90K-07, Millipore, Billerica, USA) (23). Blood was analyzed on the ADVIA 2120 within 30–60 min of collection. Serum for cytokine analysis was stored at −80°C for batch analysis at the end of the study period.

### Statistical Analysis

Statistical analysis was performed using a commercial software package (SPSS Statistics version 24, IBM, New York, NY, USA). The Shapiro Wilk test was used to assess data for normality. The data was found to be not normally distributed and therefore non-parametric statistical analysis, such as Kruskal Wallis and Mann-Whitney *U* was used to determine significance across groups; including *Babesia*-infected vs. healthy controls and non-survivors vs. survivors vs. healthy controls. Sex proportions between groups were assessed using the Chi square test. Correlations between MPXI and cytokine concentrations, as well as MPXI, and selected leukocyte counts (WBC count, band and mature neutrophil counts) were determined using the Spearman's rank correlation coefficient. The level of significance was set at *P* < 0.05. Data is represented as median and interquartile range (IQR).

## Results

### Study Population Characteristics

A total of 140 client-owned dogs naturally infected with *B. rossi* and 20 healthy control dogs from both study cohorts were included. Of the 140 *Babesia*-infected dogs included, 15 died (11%). There was a significant age difference between the groups. The median age of the *Babesia*-infected dogs (20 months; range: 2–144 months) and the survivors (20 months; range: 3–144 months) was significantly lower (*P* = 0.005 for both), than the healthy control dogs (46 months; range: 3–84 months). There was no significant age difference between the non-survivors vs. the survivors or healthy control dogs. The ratio of male to female for each group was as follows: healthy control dogs (7:13), survivors (82:43) and non-survivors (10:5), with a significant difference found between groups (*P* = 0.028).

### Comparison of MPXI and Neutrophil Counts Between Dogs With Babesiosis (Survivors and Non-survivors) and Healthy Control Dogs

**Figure 1:**
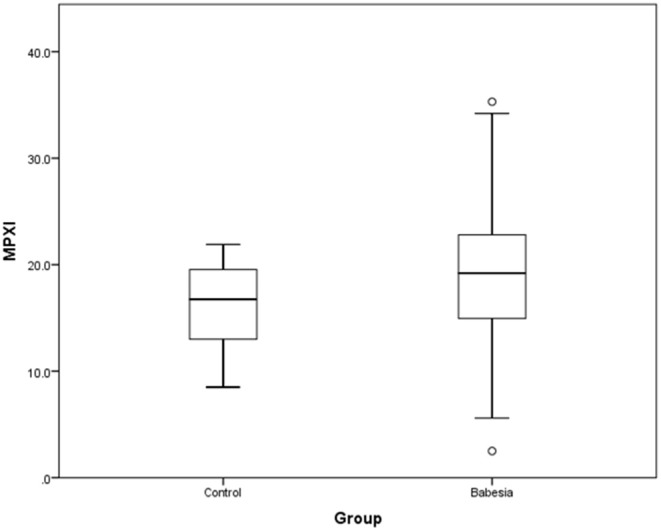
Box plot of MPXI in *B. rossi-infected* dogs (*N* = 140) at admission compared to healthy control dogs (*N* = 20). The graph displays as horizontal lines the 10th, 25th, 50th, 75th, and 90th percentiles of the MPXI. All values below the 10th percentile and above the 90th percentile are plotted separately as dots.

**Figure 2:**
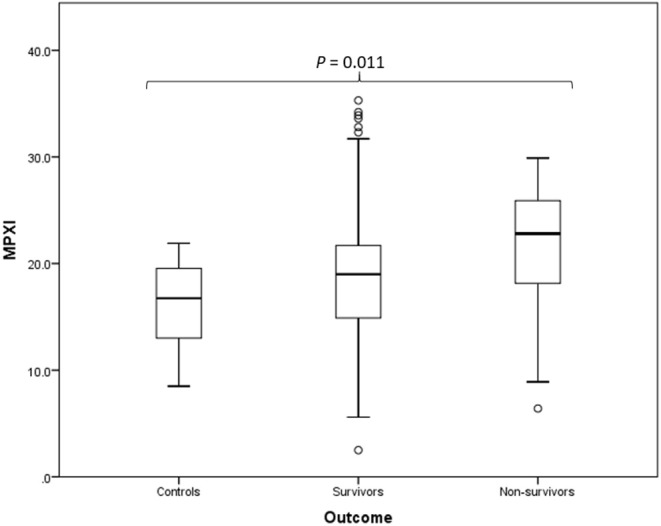
Box plot of the admission MPXI of the *B. rossi-infected* survivors (*N* = 125) and non-survivors (*N* = 15) compared to the healthy control group (*N* = 20), using Mann-Whitney *U* for statistical analysis. The graph displays as horizontal lines the 10th, 25th, 50th, 75th, and 90th percentiles of the MPXI. All values below the 10th percentile and above the 90th percentile are plotted separately as dots.

MPXI was significantly higher in the whole group of *Babesia*-infected dogs (survivors and non-survivors) (19.2; 14.9 – 22.8) compared to the healthy control dogs (16.8; 12.8 – 19.7) (*P* = 0.033) (Figure 1). MPXI was also significantly higher in the non-survivors (22.8; 17.7 – 26.1) compared to the healthy control dogs (*P* = 0.011) (Figure 2). There were no significant differences between *Babesia*-infected dogs that survived (19.0; 14.7 – 21.8) and the healthy control dogs, as well as between the survivors and non-survivors. The segmented neutrophil count (4.0 × 10^9^/L; 2.8 – 62.2) was significantly lower (*P* = 0.006) in the *Babesia*-infected dogs compared to the controls (5.8 × 10^9^/L; 5.0 – 7.5) and the band neutrophil count (0.3 × 10^9^/L; 0.2 – 0.8) significantly higher (*P* < 0.001) compared to the controls (0 × 10^9^/L; 0 – 0.1). For the *Babesia*-infected dogs that survived, the segmented neutrophil count (4.0 × 10^9^/L; 2.8 – 5.8) was significantly lower (*P* = 0.002) and the band neutrophil count (0.3 × 10^9^/L; 0.1 – 0.6) significantly higher (*P* < 0.001), compared to the controls. For the non-survivors only the band neutrophil count (1.0 × 10^9^/L; 0.5 – 1.9) was significantly higher (*P* < 0.001) compared to the controls. Both the segmented- and band neutrophil counts were significantly higher in the non-survivors compared to the survivors (*P* = 0.049 and *P* < 0.001, respectively). Results for the serum biochemistry and cytokine concentrations were previously reported for the two study cohorts and are included in the Supplementary Material (6, 7).

### Correlation of the MPXI Results With Neutrophil Counts and Various Pro-inflammatory and Immune Modulating Cytokine Concentrations

No significant correlations were found in any group between MPXI and segmented- or band neutrophil counts. Cytokine concentrations were only measured in the first study cohort and included 96 infected dogs (7). For the *Babesia*-infected group (survivors and non-survivors) a significant positive correlation was found between MPXI and IL-10 (*r* = 0.211, *P* = 0.039), and a significant negative correlation between MPXI and IL-8 (*r* = −0.350, *P* < 0.001). For the non-survivors, significant positive correlations were found between MPXI and IL-2 (*r* = 0.616*, P* = 0.033), IL-6 (*r* = 0.615, *P* = 0033), IL-18 (*r* = 0.613, *P* = 0.034), GM-CSF (*r* = 0.630*, P* = 0.028) and MCP-1 (*r* = 0.713*, P* = 0.009). For the survivors, a significant negative correlation was found between MPXI and IL-8 (*r* = −0.363, *P* = 0.001).

## Discussion

This is the first report on MPXI, as determined by the ADVIA 2120, as a marker of disease severity in dogs with *B. rossi* infection. The findings suggest that a higher MPXI is associated with a more severe inflammatory response, as determined by the concentration of pro-inflammatory cytokines, and a poorer outcome.

The MPXI was significantly higher in *B. rossi*-infected dogs, specifically the non-survivors, compared to healthy control dogs. Considering that the marked pro-inflammatory host response reported in *B. rossi* infection has been correlated with mortality (7), it was surprising, based on the current literature on MPXI changes in severe inflammation and sepsis, that the non-surviving dogs had the highest value. The expected change was that MPXI would be decreased in *Babesia*-infected dogs, especially non-survivors, due to widespread neutrophil degranulation and respiratory burst in an upregulated response to the *Babesia* parasite, as previously reported in *B. bovis* (24). Research on neutrophil function in *B. bovis* reported that neutrophils displayed increased phagocytic activity, but reduced respiratory burst during peak parasitemias (24). Moreover, studies that investigated the immune function in septic and critically ill dogs, reported that neutrophil function was impaired, possibly due to dysfunction in the NADPH oxidase complex, which is the enzyme tasked with catalyzing one of the initial reactions in the respiratory burst complex (25, 26). A concept called sepsis-induced immunosuppression or immunoparalysis, similar to what has been reported in humans, was used to describe this phenomenon and imparts a negative prognosis on affected animals (25). The presence of functional immune suppression or immunoparalysis has been previously described in complicated *B. rossi* infections secondary to increased apoptosis or redistribution of effector lymphocytes (6). MPXI data from this study were included as the second cohort of cases in our study. Additionally, highly inflammatory conditions can trigger increased granulopoiesis in the bone marrow, leading to toxic neutrophil changes. It is reported that neutrophils with toxic changes, due to accelerated maturation, contain an increased amount of MPO (19, 27, 28). With local or systemic inflammation and resultant neutrophil activation, MPO can be expected to increase, with a concomitant increase in MPXI (13). In our study, the band neutrophil count was significantly higher in the non-survivors compared to the survivors and the healthy controls. It is therefore possible that the significantly higher MPXI present in the non-survivors was not only due to increased granulopoiesis in response to an excessive inflammatory response, but was also secondary to impaired neutrophil respiratory burst function, resulting in an unutilized intracellular MPO store and a poorer prognosis in these cases.

Strong positive correlations were found between MPXI and various pro-inflammatory cytokines, including IL-2, IL-6, IL-18, GM-CSF, and MCP-1, in the dogs that died. The study from which the first cohort of cases was obtained, showed significantly higher IL-6 and MCP-1 concentrations in dogs that died (7). The study also reported that IL-2, IL-6, IL-18, and GM-CSF were significantly higher during the acute stages of *B. rossi*-infection (7). As previously discussed, increased MPXI can be in response to marked inflammation or possibly due to immunoparalysis, secondary to critical disease. A positive correlation with the above mentioned pro-inflammatory cytokines is thus expected. The previously reported significant increases in IL-6 and MCP-1 in the non-survivors might also then explain why MPXI values were the highest in the non-survivor group.

Higher MPXI in our study was correlated with lower IL-8 in survivors. IL-8 is an important moderator of neutrophil function and plays an essential role in the development of acute inflammation (29, 30). IL-8 can also inhibit neutrophil accumulation at inflammatory sites by inhibiting the adhesion of neutrophils to endothelium (30). The results of our study were interesting since our expected finding was a positive correlation between MPXI and IL-8, similar to the other pro-inflammatory cytokines. In humans with falciparum malaria, a disease with a host response reported to be similar to canine babesiosis (31), IL-8 was reported to be significantly lower in septic patients that survived, compared to the non-survivors (30). At low concentrations of IL-8, stimulation of the respiratory burst reaction might not take place, resulting in higher neutrophil MPO reserves and a resultant increased MPXI, as observed in our study. It is also possible that the decreased stimulation of the respiratory burst reaction, can result in an ineffectual pro-inflammatory response, especially in the acute phase of the disease, leading to a poorer prognosis.

A significant positive correlation was found between MPXI and the immune-modulating cytokine, IL-10, in the *Babesia*-infected dogs. Production of IL-10 seems essential in preventing an excessive inflammatory host response and thereby improving survival (32). The positive correlation seen between MPXI and IL-10 is speculated to be two-fold: Firstly, during severe sepsis, neutrophils can potentially undergo a phenotypic alteration enabling them to also produce IL-10 (33). Secondly, IL-10 is not only produced by neutrophils, but also functions to inhibit them (33, 34). An increase in neutrophil activity during sepsis could therefore result in an increased MPXI and also an increased production in IL-10. One of the ways IL-10 inhibits neutrophil function is by directly preventing respiratory burst by inhibiting NADPH oxidase (34). The inhibition of respiratory burst leads to an increase in neutrophilic MPO stores and a resultant MPXI value.

The major limitation of this study lies in its retrospective nature. The temporal differences in disease presentation could have influenced the magnitude of MPXI changes in the *B. rossi-infected* survivors and non-survivors compared to the healthy controls. Even though two study cohorts were combined to increase the size of the non-survivor group, it is still difficult to draw meaningful conclusions from this group. Multispecies software is used on the ADVIA 2120, but the representative population used for the calculation of MPXI, is human. This could have further influenced the correlation between leukocyte parameters and MPXI values. In a study looking at the predictability of disease using MPXI on the ADVIA hematology analyzer, differences in the reference values were found between breeds (11). In human studies, MPXI variation according to age and sex was found (35). The age, breed and sex heterogeneity of the study population could thus have affected the MPXI values generated. Although each peroxidase scattergram had been evaluated by an experienced clinical pathologist to confirm correct separation of the neutrophil population, MPO deficient neutrophils could still have been misclassified as monocytes. This could further have influenced the MPXI values obtained. Future standardized experimental studies to establish individual baseline values and to monitor trends in MPXI, rather than making inferences based on values taken at a single time point, will be more informative and beneficial to investigate disease pathogenesis.

In conclusion, the MPXI was significantly higher in *B. rossi-infected* dogs, specifically the dogs that died, compared to healthy control dogs. MPXI was positively correlated with the severity of the cytokine-driven pro-inflammatory host response in non-survivors and may be indicative of the presence of immunoparalysis in more severely affected dogs, leading to poorer outcome.

## Data Availability Statement

All datasets generated for this study are included in the article/Supplementary Material.

## Ethics Statement

The animal study was reviewed and approved by Faculty Research Ethics committee (REC041-18) University of Pretoria, Faculty of Veterinary Science. Written informed consent was obtained from the owners for the participation of their animals in this study.

## Author Contributions

AC: study design, data and result analysis, and primary author of the manuscript. YR: data collection for the second study cohort and manuscript editing. EH and MC: manuscript editing. AG: study design, data analysis, data collection for the first study cohort, and manuscript editing. The retrospective data was derived from studies done by AG and YR.

### Conflict of Interest

The authors declare that the research was conducted in the absence of any commercial or financial relationships that could be construed as a potential conflict of interest.

## Supplementary Material

Supplementary Table 1 Summary of the experimental data obtained from the study population.

## Abbreviations

CBC: complete blood count
CRP: C-reactive protein
GM-CSF: granulocyte-macrophage colony stimulating factor
IL: interleukin
IQR: interquartile range
MCP-1: monocyte chemo-attractant protein-1
MPO: myeloperoxidase
MPOD: myeloperoxidase deficiency
MPXI: myeloperoxidase index
NADPH: nicotinamide adenine dinucleotide phosphate
RLB-PCR: reverse line blot polymerase chain reaction
SIRS: systemic inflammatory response syndrome
TNFα: tumor necrosis factor alpha.
