# Supplementary material for: Neutrophil Myeloperoxidase Index in Dogs With Babesiosis Caused by Babesia rossi
Source: Front Vet Sci. 2020 Feb 18;7:72. doi: 10.3389/fvets.2020.00072 (PMC7040022; doi:10.3389/fvets.2020.00072)
Supplement: Supplementary Table 1 — Summary of the experimental data obtained from the study population. [file Table_1.DOCX]

| **Case** | **Age (mos)** | **Weight (kg)** | **Gender** | **Status** | **Outcome** | **WBC**  **(x10^9/L)** | **Neut Seg (x10^9/L)** | **Neut Band (x10^9/L)** | **IL-2 (pg/mL)** | **IL-6 (pg/mL)** | **IL-8 (pg/mL)** | **IL-10 (pg/mL)** | **IL-18 (pg/mL)** | **GM-CSF (pg/mL)** | **MCP-1 (pg/mL)** | **MPXI** |
| --- | --- | --- | --- | --- | --- | --- | --- | --- | --- | --- | --- | --- | --- | --- | --- | --- |
| 1 | 24 |  | Male | Infected | Survived | 4,83 | 3,14 | 0,43 | 20,3 | 53,5 | 79,5 | 532,2 | 57,0 | 27,5 | 791,4 | 32,3 |
| 2 | 24 | 14,40 | Female | Infected | Survived | 16,33 | 10,45 | 2,29 | 31,9 | 62,9 | 6186,0 | 244,1 | 66,3 | 29,2 | 314,5 | 19,8 |
| 3 | 72 | 11,00 | Male | Infected | Survived | 5,64 | 4,40 | 0,34 | 617,3 | 569,1 | 1393,2 | 437,3 | 1766,6 | 492,9 | 667,0 | 16,9 |
| 4 | 15 | 11,00 | Male | Infected | Survived | 4,11 | 3,04 | 0,08 | 3,4 | 28,1 | 125,7 | 4762,5 | 32,4 | 15,6 | 315,5 | 27,4 |
| 5 | 48 |  | Female | Infected | Survived | 4,46 | 2,99 | 0,04 | 69,7 | 138,4 | 45,1 | 1167,5 | 339,1 | 133,4 | 519,8 | 31,7 |
| 6 | 36 | 22,80 | Male | Infected | Survived | 17,61 | 12,33 | 1,58 | 13,1 | 26,5 | 1310,4 | 391,5 | 29,9 | 13,9 | 348,5 | 34,2 |
| 7 | 19 |  | Female | Infected | Survived | 11,09 | 5,77 | 0,22 | 12,2 | 21,8 | 687,2 | 1833,6 | 48,7 | 18,2 | 198,9 | 21,7 |
| 8 | 12 |  | Male | Infected | Survived | 8,98 | 5,75 | 0,00 | 29,2 | 121,1 | 133,4 | 370,2 | 129,5 | 61,0 | 673,0 | 35,3 |
| 9 | 7 |  | Male | Infected | Survived | 5,82 | 3,72 | 0,17 | 3,4 | 28,1 | 21,6 | 4756,6 | 22,3 | 9,1 | 424,1 | 32,8 |
| 10 | 24 |  | Female | Infected | Survived | 4,39 | 2,33 | 0,31 | 92,4 | 128,9 | 141,1 | 1535,9 | 231,9 | 159,8 | 527,6 | 33,6 |
| 11 | 4 | 8,4 | Male | Infected | Survived | 15,22 | 9,59 | 2,28 | 22,0 | 133,7 | 5488,8 | 332,5 | 48,7 | 25,0 | 293,9 | 33,9 |
| 12 | 10 | 26,0 | Female | Infected | Survived | 6,63 | 4,77 | 0,07 | 3,4 | 29,7 | 8748,6 | 432,4 | 17,3 | 9,1 | 262,6 | 18,6 |
| 13 | 79 | 40,4 | Male | Infected | Died | 16,64 | 9,48 | 1,66 | 3,4 | 25,0 | 21,6 | 951,5 | 5,7 | 9,1 | 620,7 | 22,7 |
| 14 | 6 | 6,8 | Female | Infected | Survived | 21,06 | 17,06 | 0,42 | 3,4 | 39,3 | 3561,9 | 334,2 | 18,5 | 9,1 | 198,2 | 25,2 |
| 15 | 20 | 15,0 | Male | Infected | Survived | 6,66 | 4,33 | 0,13 | 45,2 | 171,4 | 2404,1 | 8189,7 | 169,3 | 110,4 | 613,6 | 29,4 |
| 16 | 9 | 29,8 | Male | Infected | Survived | 7,79 | 6,15 | 0,31 | 2529,7 | 9315,4 | 292,9 | 437,3 | 745,1 | 1039,9 | 1854,0 | 28,5 |
| 17 | 12 | 35,0 | Male | Infected | Survived | 6,36 | 3,24 | 0,19 | 135,9 | 268,3 | 3376,8 | 381,7 | 163,4 | 85,3 | 415,9 | 20,0 |
| 18 | 12 | 4,1 | Male | Infected | Survived | 2,90 | 1,39 | 0,17 | 34,5 | 56,6 | 3117,8 | 1134,8 | 86,2 | 19,0 | 358,5 | 17,7 |
| 19 | 53 | 33,0 | Female | Infected | Survived | 8,54 | 4,36 | 2,56 | 34,5 | 94,4 | 57,3 | 811,8 | 52,3 | 40,3 | 742,1 | 14,0 |
| 20 | 6 | 10,4 | Female | Infected | Survived | 7,87 | 4,09 | 0,31 | 74,2 | 165,1 | 2313,8 | 128,7 | 124,8 | 112,4 | 293,5 | 17,7 |
| 21 | 2 |  | Female | Infected | Died | 5,78 | 3,47 | 0,46 | 53,4 | 86,5 | 791,8 | 1590,3 | 82,7 | 62,3 | 657,1 | 25,7 |
| 22 | 10 | 25,0 | Male | Infected | Survived | 11,22 | 8,53 | 1,01 | 19,4 | 55,1 | 2577,1 | 162,7 | 43,7 | 20,7 | 211,4 | 16,8 |
| 23 | 32 | 10,8 | Male | Infected | Survived | 3,73 | 2,54 | 0,45 | 2389,8 | 6718,3 | 5639,0 | 12786,5 | 2249,8 | 2920,8 | 5950,5 | 28,7 |
| 24 | 84 | 65,0 | Male | Infected | Survived | 16,60 | 10,62 | 1,83 | 53,4 | 64,5 | 10065,4 | 628,8 | 97,9 | 75,9 | 307,0 | 16,1 |
| 25 | 3 | 10,4 | Female | Infected | Survived | 7,44 | 2,98 | 0,00 | 3,4 | 18,6 | 857,8 | 412,8 | 16,0 | 9,1 | 769,0 | 14,9 |
| 26 | 3 | 11,4 | Male | Infected | Survived | 7,38 | 4,72 | 0,07 | 3,4 | 29,7 | 1964,7 | 969,7 | 32,4 | 9,1 | 800,2 | 20,3 |
| 27 | 7 | 14,0 | Male | Infected | Survived | 4,63 | 2,64 | 0,14 | 136,8 | 241,8 | 834,7 | 851,2 | 110,8 | 119,9 | 330,1 | 17,4 |
| 28 | 12 | 13,4 | Male | Infected | Survived | 5,19 | 3,32 | 0,26 | 273,2 | 495,8 | 171,9 | 3019,6 | 306,1 | 204,4 | 459,5 | 21,8 |
| 29 | 84 | 30,0 | Female | Infected | Survived | 5,90 | 4,78 | 0,06 | 37,2 | 99,1 | 21,6 | 555,2 | 31,2 | 43,7 | 563,7 | 17,0 |
| 30 | 48 | 49,6 | Male | Infected | Survived | 4,29 | 1,89 | 0,69 | 39,9 | 77,1 | 1097,2 | 607,5 | 67,5 | 54,6 | 372,3 | 28,2 |
| 31 | 7 | 16,6 | Male | Infected | Survived | 5,78 | 3,76 | 0,23 | 3,4 | 3,6 | 880,8 | 906,2 | 24,8 | 9,1 | 323,7 | 16,5 |
| 32 | 48 | 20,0 | Female | Infected | Survived | 6,07 | 4,49 | 0,18 | 84,2 | 121,1 | 3610,1 | 470,0 | 166,9 | 75,9 | 555,9 | 17,3 |
| 33 | 18 | 32,0 | Male | Infected | Survived | 6,08 | 3,71 | 0,00 | 3,4 | 3,6 | 256,7 | 781,8 | 24,8 | 9,1 | 203,3 | 15,9 |
| 34 | 4 | 18,4 | Male | Infected | Died | 6,73 | 4,04 | 0,54 | 363,9 | 1925,9 | 2737,8 | 3702,9 | 981,2 | 640,5 | 1665,1 | 27,4 |
| 35 | 32 | 33,0 | Male | Infected | Survived | 9,04 | 4,70 | 2,35 | 15,9 | 73,2 | 4610,1 | 961,2 | 36,6 | 9,1 | 527,4 | 20,6 |
| 36 | 28 | 9,0 | Male | Infected | Survived | 5,32 | 2,77 | 1,38 | 164,7 | 218,2 | 447,3 | 228,5 | 587,9 | 263,8 | 544,2 | 23,8 |
| 37 | 35 | 13,0 | Male | Infected | Died | 17,78 | 11,02 | 3,20 | 3,4 | 788,0 | 2627,9 | 303,5 | 5,7 | 9,1 | 500,0 | 11,0 |
| 38 | 24 | 25,8 | Male | Infected | Survived | 5,60 | 3,47 | 0,50 | 14,0 | 19,9 | 754,1 | 93,2 | 45,1 | 20,3 | 230,8 | 19,2 |
| 39 | 96 | 37,0 | Female | Infected | Survived | 5,91 | 5,44 | 0,12 | 103,4 | 229,1 | 111,4 | 512,8 | 142,1 | 117,6 | 700,6 | 22,3 |
| 40 | 23 | 8,0 | Male | Infected | Survived | 5,19 | 3,22 | 0,16 | 3,4 | 3,6 | 5693,8 | 197,4 | 22,7 | 9,1 | 322,9 | 13,7 |
| 41 | 24 | 7,2 | Female | Infected | Survived | 3,68 | 2,28 | 0,26 | 43,3 | 57,4 | 101,0 | 169,7 | 80,6 | 44,3 | 360,7 | 25,4 |
| 42 | 144 | 18,2 | Male | Infected | Survived | 5,91 | 4,43 | 0,00 | 3,4 | 3,6 | 686,4 | 938,4 | 33,1 | 9,1 | 485,0 | 17,2 |
| 43 | 8 | 22,0 | Male | Infected | Survived | 11,49 | 4,25 | 3,91 | 15,0 | 540,6 | 9608,0 | 97,3 | 43,6 | 18,5 | 687,8 | 14,1 |
| 44 | 96 | 3,0 | Female | Infected | Survived | 6,22 | 4,48 | 0,12 | 26,9 | 143,4 | 1066,3 | 889,4 | 49,5 | 22,0 | 1484,3 | 25,8 |
| 45 | 120 | 54,0 | Male | Infected | Survived | 6,21 | 4,04 | 0,37 | 3,4 | 3,6 | 50,1 | 140,8 | 31,4 | 9,1 | 327,5 | 26,7 |
| 46 | 9 | 28,0 | Male | Infected | Survived | 6,43 | 4,31 | 0,32 | 261,5 | 520,5 | 838,1 | 276,1 | 396,1 | 296,3 | 459,4 | 15,2 |
| 47 | 78 | 9,2 | Male | Infected | Survived | 3,06 | 1,53 | 0,12 | 3,4 | 29,6 | 60,5 | 1799,5 | 31,4 | 9,1 | 402,1 | 21,4 |
| 48 | 11 | 19,8 | Male | Infected | Survived | 3,80 | 1,06 | 1,44 | 16,0 | 94,1 | 229,1 | 2191,9 | 35,7 | 17,9 | 1470,9 | 21,2 |
| 49 | 20 | 15,6 | Male | Infected | Survived | 12,88 | 11,08 | 0,90 | 3,4 | 46,2 | 1188,0 | 325,5 | 22,7 | 9,1 | 883,9 | 25,5 |
| 50 | 35 | 33,2 | Male | Infected | Survived | 33,12 | 20,53 | 2,65 | 766,1 | 549,5 | 1054,8 | 656,7 | 1097,0 | 410,1 | 535,0 | 15,9 |
| 51 | 21 | 4,4 | Female | Infected | Survived | 5,18 | 3,06 | 0,41 | 98,0 | 159,7 | 1851,5 | 78,0 | 97,8 | 94,9 | 539,8 | 17,3 |
| 52 | 18 | 36,0 | Female | Infected | Survived | 11,36 | 9,09 | 0,11 | 17,6 | 35,7 | 859,5 | 176,5 | 28,8 | 19,8 | 651,8 | 16,6 |
| 53 | 54 | 14,2 | Male | Infected | Survived | 13,67 | 7,11 | 2,32 | 36,6 | 74,0 | 300,8 | 150,1 | 25,3 | 26,7 | 395,4 | 20,6 |
| 54 | 10 |  | Male | Infected | Survived | 8,25 | 4,95 | 0,58 | 86,6 | 391,4 | 682,4 | 776,3 | 128,7 | 133,1 | 1218,0 | 27,8 |
| 55 | 6 | 3,0 | Female | Infected | Died | 5,06 | 3,14 | 0,71 | 652,4 | 1022,3 | 776,2 | 366,9 | 734,3 | 515,3 | 1017,1 | 27,0 |
| 56 |  | 7,0 | Female | Infected | Survived | 10,01 | 7,01 | 1,00 | 23,7 | 48,8 | 9135,9 | 633,5 | 46,2 | 24,2 | 426,6 | 13,6 |
| 57 | 4 | 13,8 | Male | Infected | Survived | 6,16 | 4,74 | 0,06 | 1342,2 | 2300,3 | 1361,0 | 1931,6 | 616,1 | 568,0 | 1424,2 | 19,5 |
| 58 | 12 | 21,6 | Male | Infected | Died | 14,75 | 11,21 | 1,77 | 21,4 | 971,5 | 1295,9 | 830,9 | 26,2 | 16,0 | 1284,0 | 22,8 |
| 59 | 4 | 8,0 | Male | Infected | Died | 4,62 | 3,42 | 0,18 | 55,6 | 341,3 | 1662,4 | 2183,4 | 236,9 | 133,7 | 1194,7 | 21,4 |
| 60 | 40 | 7,5 | Male | Infected | Survived | 2,30 | 1,59 | 0,07 | 38,9 | 82,8 | 880,2 | 1934,4 | 101,4 | 93,8 | 619,1 | 26,8 |
| 61 | 37 | 26,2 | Male | Infected | Died | 23,93 | 16,99 | 1,91 | 992,9 | 2110,0 | 1907,6 | 330,5 | 1338,8 | 733,9 | 1380,2 | 29,9 |
| 62 | 5 | 17,8 | Female | Infected | Survived | 24,67 | 17,27 | 2,47 | 25,2 | 92,9 | 685,8 | 130,6 | 35,7 | 19,2 | 690,7 | 21,6 |
| 63 | 38 | 21,6 | Female | Infected | Survived | 4,75 | 3,14 | 0,00 | 1606,2 | 3022,4 | 2323,8 | 1378,0 | 2800,2 | 736,4 | 1523,7 | 16,7 |
| 64 | 56 | 28,2 | Female | Infected | Survived | 20,49 | 10,86 | 2,25 | 13,7 | 35,7 | 4005,7 | 105,3 | 31,4 | 19,8 | 283,1 | 21,1 |
| 65 | 72 | 27,0 | Female | Infected | Died | 25,70 | 16,96 | 1,80 | 3,4 | 74,0 | 1047,4 | 309,0 | 20,1 | 9,1 | 826,3 | 26,1 |
| 66 | 132 | 5,8 | Female | Infected | Survived | 3,86 | 2,70 | 0,23 | 375,3 | 663,3 | 362,2 | 48,8 | 865,6 | 410,1 | 1318,8 | 20,5 |
| 67 | 3 | 4,6 | Female | Infected | Survived | 1,80 | 0,14 | 0,22 | 29,0 | 516,7 | 11817,4 | 1117,3 | 51,5 | 33,7 | 1819,9 | 19,2 |
| 68 | 9 | 24,2 | Female | Infected | Survived | 9,78 | 5,77 | 0,78 | 3,4 | 26,6 | 2598,3 | 754,5 | 44,4 | 24,2 | 568,8 | 19,1 |
| 69 | 9 | 5,2 | Male | Infected | Survived | 6,21 | 3,35 | 0,56 | 3,4 | 3,6 | 3300,5 | 219,8 | 22,7 | 9,1 | 418,5 | 19,3 |
| 70 | 11 | 12,0 | Female | Infected | Survived | 7,35 | 4,63 | 0,51 | 3,4 | 3,6 | 239,4 | 141,4 | 20,9 | 9,1 | 396,8 | 23,3 |
| 71 | 4 | 12,6 | Male | Infected | Survived | 7,85 | 5,57 | 0,31 | 3,4 | 3,6 | 4811,9 | 653,4 | 15,7 | 9,1 | 740,3 | 8,7 |
| 72 | 42 | 23,2 | Male | Infected | Survived | 16,80 | 12,26 | 0,34 | 3,4 | 53,8 | 2391,7 | 491,0 | 14,8 | 9,1 | 434,7 | 18,7 |
| 73 | 96 | 40,0 | Male | Infected | Survived | 5,87 | 3,52 | 0,18 | 3,4 | 38,4 | 1377,2 | 875,1 | 33,1 | 19,8 | 362,6 | 16,0 |
| 74 | 36 | 20,0 | Male | Infected | Survived | 4,37 | 2,84 | 0,00 | 35,9 | 84,1 | 30,5 | 510,9 | 66,0 | 63,4 | 455,3 | 20,4 |
| 75 | 18 | 50,0 | Male | Infected | Survived | 12,73 | 6,87 | 0,64 | 61,7 | 110,5 | 4437,8 | 304,0 | 115,9 | 82,0 | 424,4 | 12,1 |
| 76 | 24 | 12,0 | Male | Infected | Survived | 6,34 | 3,49 | 0,32 | 27,5 | 41,0 | 410,0 | 365,2 | 72,4 | 42,5 | 368,1 | 22,9 |
| 77 | 13 | 29,4 | Male | Infected | Survived | 36,00 | 24,48 | 5,76 | 16,0 | 401,0 | 1118,4 | 340,4 | 32,3 | 16,0 | 1253,8 |  |
| 78 | 7 | 31,6 | Female | Infected | Survived | 7,14 | 5,14 | 0,00 | 3,4 | 13,5 | 3029,1 | 251,1 | 20,9 | 9,1 | 587,2 | 21,2 |
| 79 | 108 | 35,6 | Male | Infected | Survived | 4,69 | 2,81 | 0,47 | 22,9 | 61,2 | 1377,2 | 620,3 | 41,8 | 21,7 | 709,0 | 13,2 |
| 80 | 8 | 28,2 | Female | Infected | Survived | 6,29 | 3,96 | 0,00 | 50001,0 | 50001,0 | 1677,8 | 937,2 | 50001,0 | 50001,0 | 7974,3 | 20,5 |
| 81 | 29 | 12,8 | Female | Infected | Died | 4,30 | 1,94 | 0,73 | 3,4 | 13,8 | 8861,0 | 576,8 | 22,5 | 9,1 | 472,9 | 8,9 |
| 82 | 24 | 38,0 | Male | Infected | Survived | 4,31 | 3,15 | 0,30 | 546,3 | 860,0 | 840,0 | 837,5 | 1625,4 | 720,2 | 927,9 | 11,2 |
| 83 | 96 | 19,0 | Male | Infected | Survived | 19,09 | 13,55 | 3,25 | 16,0 | 91,2 | 36889,7 | 747,9 | 53,0 | 69,8 | 1021,6 | 25,6 |
| 84 | 9 |  | Female | Infected | Died | 4,12 | 0,91 | 0,99 | 28,2 | 1122,4 | 1008,4 | 2680,1 | 47,4 | 29,3 | 1690,2 | 25,6 |
| 85 | 36 | 18,6 | Male | Infected | Died | 4,99 | 3,79 | 0,40 | 165,5 | 499,7 | 242,6 | 867,6 | 182,3 | 113,1 | 563,1 | 18,6 |
| 86 | 96 | 38,0 | Male | Infected | Survived | 5,69 | 4,04 | 0,11 | 174,1 | 835,5 | 385,3 | 1240,4 | 471,2 | 257,2 | 1815,7 | 19,9 |
| 87 | 12 | 20,0 | Male | Infected | Survived | 13,08 | 8,24 | 1,05 | 3,4 | 910,9 | 7450,7 | 4571,4 | 19,1 | 9,1 | 664,7 | 20,5 |
| 88 | 36 | 9,0 | Male | Infected | Survived | 3,34 | 2,47 | 0,20 | 1256,8 | 2690,7 | 68,8 | 357,1 | 1792,4 | 1181,5 | 1283,0 | 28,2 |
| 89 | 24 | 25,6 | Female | Infected | Survived | 6,38 | 4,72 | 0,06 | 3,4 | 3,6 | 321,9 | 768,5 | 20,2 | 9,1 | 365,7 | 19,5 |
| 90 | 12 | 14,2 | Male | Infected | Survived | 5,56 | 3,06 | 0,78 | 2716,1 | 3599,7 | 2287,5 | 2840,3 | 4936,7 | 2446,3 | 707,6 | 29,2 |
| 91 | 36 | 6,6 | Female | Infected | Survived | 4,72 | 2,55 | 0,47 | 112,4 | 135,0 | 551,6 | 1669,9 | 175,9 | 127,1 | 537,9 | 26,9 |
| 92 | 3 | 8,6 | Male | Infected | Survived | 3,06 | 0,92 | 0,24 | 3,4 | 47,2 | 314,0 | 1673,6 | 23,7 | 13,0 | 732,0 | 21,1 |
| 93 | 6 | 20,2 | Male | Infected | Survived | 10,90 | 6,00 | 0,98 | 34,7 | 60,5 | 5686,1 | 313,4 | 66,8 | 38,2 | 325,8 | 19,0 |
| 94 | 11 | 55,0 | Male | Infected | Survived | 13,94 | 7,81 | 0,14 | 27,2 | 53,2 | 1252,4 | 325,9 | 56,1 | 33,4 | 149,6 | 21,8 |
| 95 | 10 | 20,0 | Female | Infected | Survived | 3,62 | 2,24 | 0,04 | 445,6 | 469,4 | 27723,4 | 846,9 | 680,7 | 302,9 | 391,9 | 12,8 |
| 96 | 36 | 10,0 | Male | Infected | Survived | 3,58 | 1,00 | 0,14 | 13768,3 | 19018,6 | 567,5 | 1519,2 | 4052,2 | 6412,9 | 2450,5 | 19,6 |
| 97 | 12 | 28,8 | Male | Infected | Survived | 6,67 | 4,54 | 0,13 | 14,1 | 102,9 | 804,6 | 140,0 | 39,7 | 18,7 | 501,9 | 18,5 |
| 101 | 23 | 19,3 | Male | Infected | Survived | 6,27 | 4,45 | 0,50 |  |  |  |  |  |  |  | 10,9 |
| 102 | 11 | 28,6 | Male | Infected | Survived | 5,03 | 3,07 | 0,15 |  |  |  |  |  |  |  | 7,6 |
| 103 | 9 | 11,8 | Female | Infected | Survived | 6,38 | 4,47 | 0,57 |  |  |  |  |  |  |  | 18,3 |
| 104 | 9 | 4,7 | Male | Infected | Survived | 5,30 | 1,86 | 1,38 |  |  |  |  |  |  |  | 13,3 |
| 105 | 48 | 18,0 | Female | Infected | Survived | 7,14 | 5,43 | 0,43 |  |  |  |  |  |  |  | 14,0 |
| 106 | 43 | 9,8 | Male | Infected | Survived | 5,27 | 3,27 | 0,00 |  |  |  |  |  |  |  | 15,8 |
| 107 | 60 | 7,4 | Male | Infected | Survived | 2,55 | 1,89 | 0,15 |  |  |  |  |  |  |  | 21,0 |
| 108 | 24 | 11,6 | Male | Infected | Survived | 11,63 | 6,28 | 2,44 |  |  |  |  |  |  |  | 15,8 |
| 109 | 36 | 32,0 | Male | Infected | Survived | 6,01 | 4,39 | 0,24 |  |  |  |  |  |  |  | 16,5 |
| 110 | 6 | 23,0 | Female | Infected | Survived | 6,53 | 3,85 | 0,33 |  |  |  |  |  |  |  | 21,0 |
| 111 | 9 | 18,6 | Male | Infected | Survived | 5,58 | 3,29 | 1,28 |  |  |  |  |  |  |  | 11,4 |
| 112 | 3 | 8,8 | Female | Infected | Survived | 23,60 | 20,53 | 0,24 |  |  |  |  |  |  |  | 17,3 |
| 113 | 18 | 6,8 | Male | Infected | Survived | 1,99 | 0,44 | 0,52 |  |  |  |  |  |  |  | 17,3 |
| 114 | 19 | 23,8 | Male | Infected | Survived | 4,75 | 2,76 | 0,81 |  |  |  |  |  |  |  | 19,7 |
| 115 | 5 | 5,8 | Female | Infected | Survived | 3,20 | 2,37 | 0,13 |  |  |  |  |  |  |  | 22,8 |
| 116 | 6 | 12,7 | Male | Infected | Survived | 6,36 | 4,07 | 0,13 |  |  |  |  |  |  |  | 18,8 |
| 117 | 36 | 14,6 | Male | Infected | Survived | 10,21 | 7,66 | 0,31 |  |  |  |  |  |  |  | 7,6 |
| 118 | 24 | 14,7 | Male | Infected | Survived | 11,50 | 5,87 | 0,69 |  |  |  |  |  |  |  | 11,1 |
| 119 | 48 | 31,6 | Male | Infected | Survived | 5,02 | 2,81 | 0,25 |  |  |  |  |  |  |  | 15,0 |
| 120 | 72 | 10,6 | Male | Infected | Survived | 7,56 | 5,14 | 0,30 |  |  |  |  |  |  |  | 13,9 |
| 121 | 10 | 3,4 | Female | Infected | Survived | 4,20 | 1,76 | 0,59 |  |  |  |  |  |  |  | 12,7 |
| 122 | 10 | 7,8 | Female | Infected | Survived | 3,90 | 2,65 | 0,39 |  |  |  |  |  |  |  | 10,8 |
| 123 | 4 | 3,6 | Female | Infected | Survived | 3,76 | 3,24 | 0,25 |  |  |  |  |  |  |  | 14,1 |
| 124 | 18 | 6,4 | Male | Infected | Survived | 3,63 | 2,87 | 0,15 |  |  |  |  |  |  |  | 13,2 |
| 125 | 72 | 6,2 | Male | Infected | Survived | 5,80 | 2,32 | 0,99 |  |  |  |  |  |  |  | 5,6 |
| 126 | 30 | 30,6 | Male | Infected | Died | 103,55 | 67,31 | 21,75 |  |  |  |  |  |  |  | 6,4 |
| 127 | 9 | 45,6 | Male | Infected | Died | 21,60 | 15,12 | 0,86 |  |  |  |  |  |  |  | 23,7 |
| 128 | 23 | 20,0 | Female | Infected | Survived | 9,94 | 8,35 | 0,40 |  |  |  |  |  |  |  | 20,3 |
| 129 | 29 | 21,0 | Male | Infected | Survived | 8,81 | 5,81 | 0,44 |  |  |  |  |  |  |  | 16,2 |
| 130 | 36 | 6,5 | Female | Infected | Survived | 1,12 | 0,66 | 0,09 |  |  |  |  |  |  |  | 13,0 |
| 131 | 60 | 34,0 | Male | Infected | Died | 14,36 | 9,19 | 2,87 |  |  |  |  |  |  |  | 17,7 |
| 132 | 10 | 26,7 | Male | Infected | Survived | 5,20 | 3,95 | 0,16 |  |  |  |  |  |  |  | 9,6 |
| 133 | 30 | 29,0 | Female | Infected | Survived | 7,11 | 4,55 | 0,36 |  |  |  |  |  |  |  | 19,9 |
| 134 | 18 | 6,0 | Female | Infected | Survived | 9,63 | 8,28 | 0,10 |  |  |  |  |  |  |  | 19,8 |
| 135 | 12 | 10,6 | Female | Infected | Survived | 5,14 | 2,26 | 0,26 |  |  |  |  |  |  |  | 26,4 |
| 136 | 3 | 11,5 | Male | Infected | Survived | 9,27 | 7,05 | 0,65 |  |  |  |  |  |  |  | 20,6 |
| 137 | 42 | 5,6 | Male | Infected | Survived | 5,40 | 2,16 | 0,54 |  |  |  |  |  |  |  | 13,9 |
| 138 | 91 | 9,8 | Male | Infected | Survived | 5,19 | 2,39 | 0,42 |  |  |  |  |  |  |  | 15,1 |
| 139 | 10 | 2,6 | Male | Infected | Survived | 4,90 | 2,65 | 0,39 |  |  |  |  |  |  |  | 8,9 |
| 140 | 84 | 8,6 | Male | Infected | Survived | 4,04 | 2,10 | 0,00 |  |  |  |  |  |  |  | 14,5 |
| 141 | 11 | 11,6 | Male | Infected | Survived | 9,59 | 7,10 | 0,48 |  |  |  |  |  |  |  | 2,5 |
| 142 | 8 | 12,6 | Male | Infected | Survived | 12,81 | 7,69 | 2,18 |  |  |  |  |  |  |  | 6,9 |
| 143 | 24 | 6,8 | Female | Infected | Survived | 4,47 | 2,99 | 0,18 |  |  |  |  |  |  |  | 13,3 |
| 144 | 20 | 8,8 | Male | Infected | Survived | 4,84 | 1,94 | 0,19 |  |  |  |  |  |  |  | 13,6 |
| 201 | 24 | 40,6 | Male | Control | Control | 14,07 | 9,29 | 0,00 | 12,2 | 42,4 | 1655,3 | 8,4 | 34,9 | 18,2 | 115,0 | 19,9 |
| 202 | 84 | 34,0 | Female | Control | Control | 8,58 | 5,83 | 0,00 | 227,3 | 291,7 | 7898,4 | 401,3 | 851,9 | 387,7 | 278,9 | 19,2 |
| 203 | 72 | 31,0 | Female | Control | Control | 7,80 | 4,91 | 0,00 | 4162,6 | 4393,4 | 1528,1 | 19,5 | 5241,0 | 2501,7 | 334,0 | 20,7 |
| 204 | 84 | 23,6 | Male | Control | Control | 12,22 | 8,55 | 0,24 | 3,4 | 16,1 | 837,3 | 1015,8 | 15,7 | 16,6 | 20,9 | 20,0 |
| 205 | 24 | 30,0 | Female | Control | Control | 11,60 | 5,80 | 0,00 | 16,0 | 43,6 | 2930,3 | 56,6 | 32,3 | 27,4 | 234,5 | 12,5 |
| 206 | 84 |  | Female | Control | Control | 8,05 | 5,31 | 0,08 | 34,3 | 68,9 | 3769,1 | 280,9 | 43,6 | 21,1 | 208,6 | 21,9 |
| 207 | 48 | 15,0 | Female | Control | Control | 8,99 | 4,14 | 0,00 | 13,1 | 23,4 | 4063,1 | 6612,4 | 24,8 | 9,1 | 174,2 | 16,0 |
| 208 | 64 | 8,8 | Female | Control | Control | 10,57 | 7,50 | 0,11 | 405,5 | 663,1 | 4886,1 | 8,4 | 367,1 | 9,1 | 275,3 | 20,4 |
| 209 | 78 | 35,0 | Male | Control | Control | 4,72 | 3,12 | 0,00 | 3,4 | 3,6 | 1291,1 | 8,4 | 5,7 | 9,1 | 94,5 | 19,1 |
| 210 | 84 | 27,0 | Female | Control | Control | 10,84 | 7,59 | 0,11 | 12,2 | 26,5 | 6033,5 | 30,5 | 33,7 | 34,5 | 103,8 | 17,7 |
| 211 | 18 | 65,0 | Male | Control | Control | 7,86 | 3,54 | 0,24 | 937,0 | 1355,5 | 1261,0 | 8,4 | 1393,5 | 783,3 | 270,7 | 13,5 |
| 212 | 3 | 9,0 | Male | Control | Control | 17,23 | 8,96 | 0,17 | 19,0 | 29,7 | 5810,5 | 135,9 | 53,1 | 21,8 | 211,7 | 10,0 |
| 213 | 3 | 8,0 | Female | Control | Control | 13,28 | 6,11 | 0,00 | 168,2 | 189,3 | 3998,5 | 95,5 | 264,1 | 166,3 | 321,2 | 10,4 |
| 214 | 27 | 29,0 | Female | Control | Control | 8,39 | 4,20 | 0,08 | 54,0 | 348,3 | 3316,7 | 8,4 | 420,1 | 125,8 | 222,6 | 15,2 |
| 215 | 24 | 23,0 | Female | Control | Control | 11,46 | 5,50 | 0,00 | 3,4 | 3,6 | 1767,2 | 44,6 | 5,7 | 9,1 | 160,3 | 17,5 |
| 216 | 76 | 30,9 | Male | Control | Control | 7,68 | 5,22 | 0,00 |  |  |  |  |  |  |  | 8,5 |
| 217 | 72 | 35,0 | Female | Control | Control | 10,84 | 6,83 | 0,11 |  |  |  |  |  |  |  | 18,7 |
| 218 | 44 | 30,0 | Female | Control | Control | 14,43 | 7,50 | 0,00 |  |  |  |  |  |  |  | 14,0 |
| 219 | 24 | 20,0 | Male | Control | Control | 9,21 | 5,16 | 0,00 |  |  |  |  |  |  |  | 11,1 |
| 220 | 42 | 25,0 | Female | Control | Control | 8,25 | 5,78 | 0,00 |  |  |  |  |  |  |  | 15,8 |
